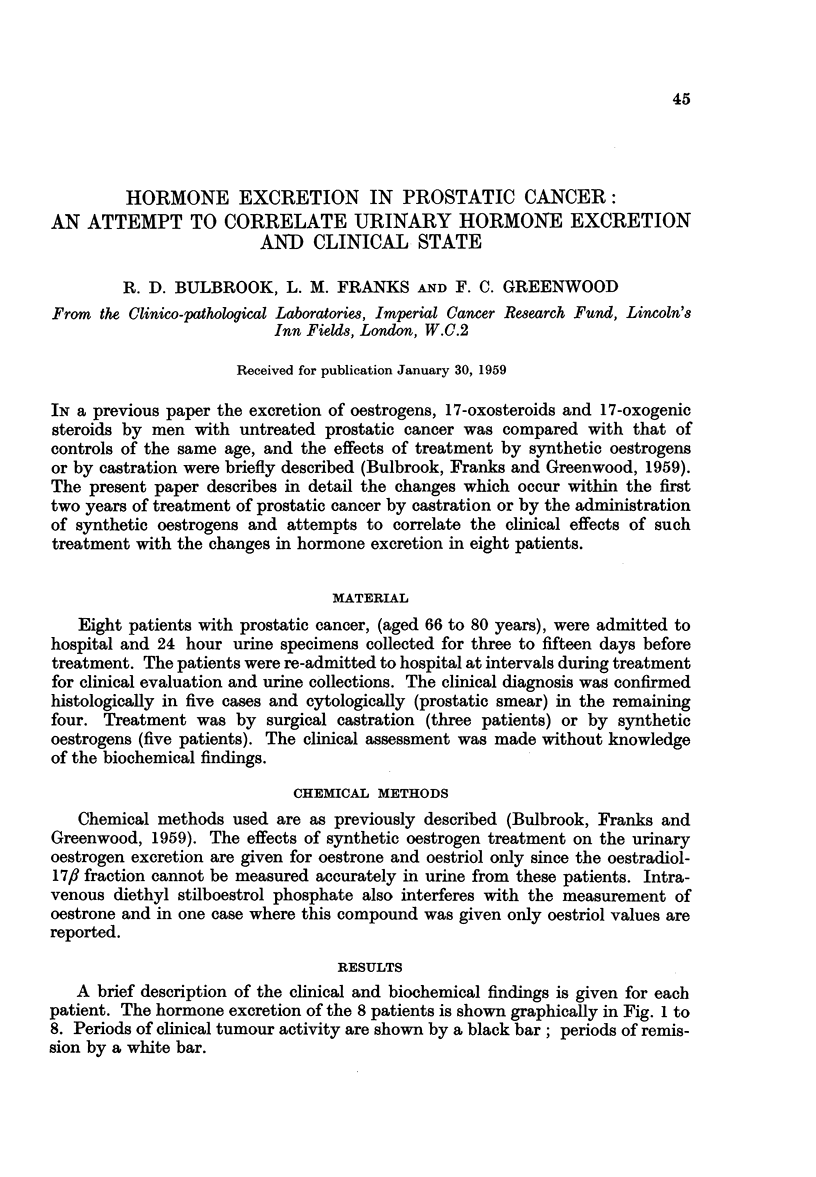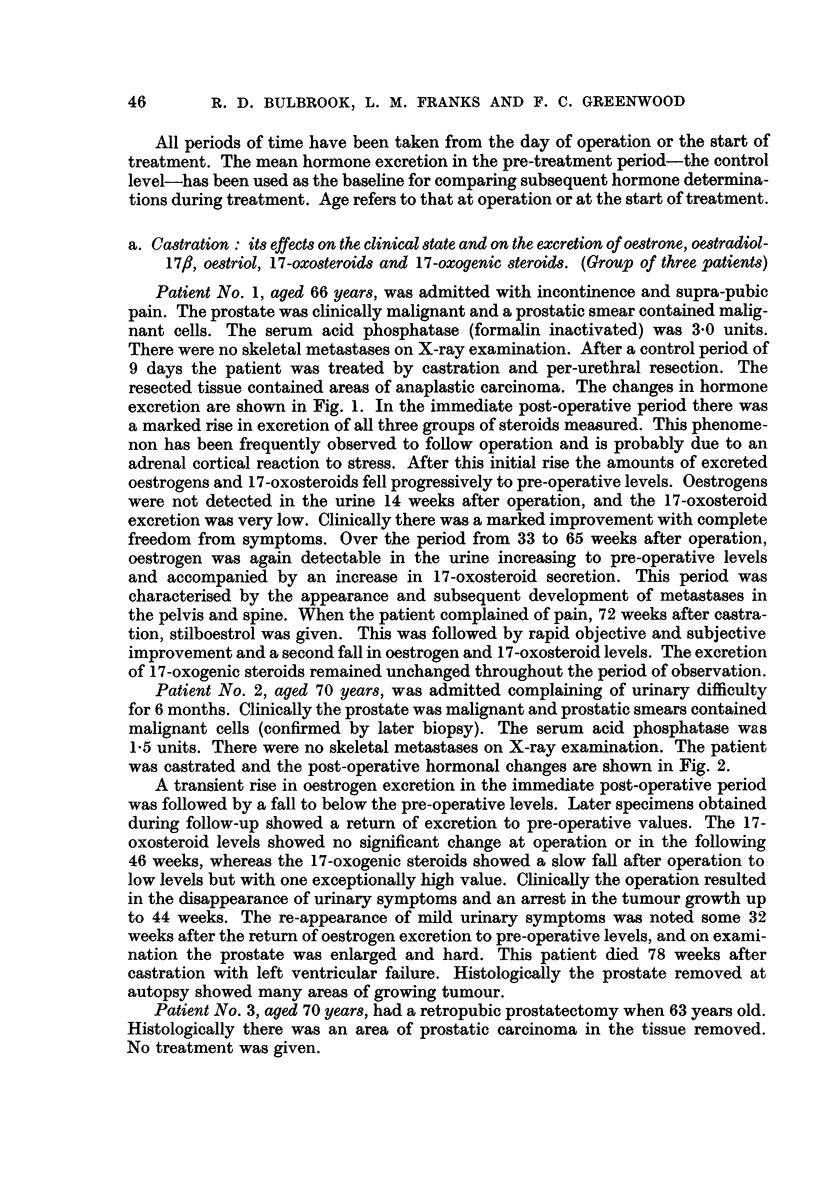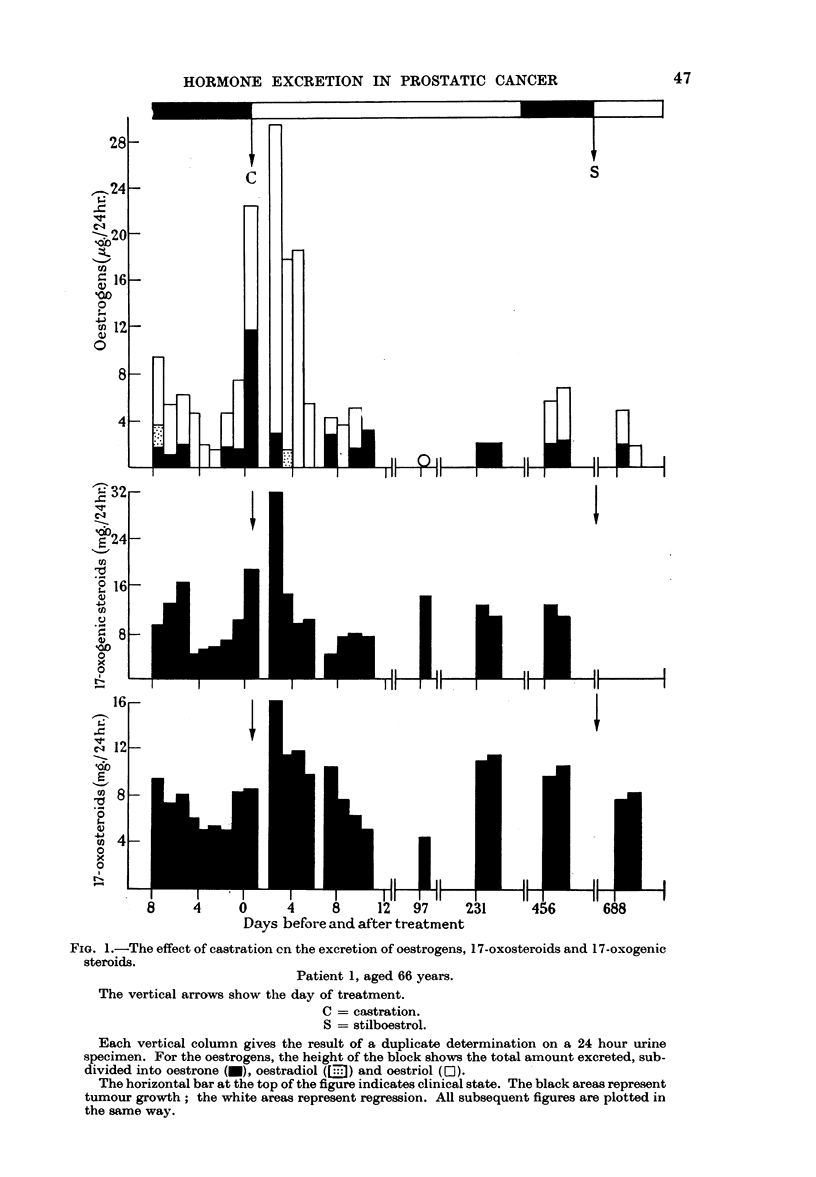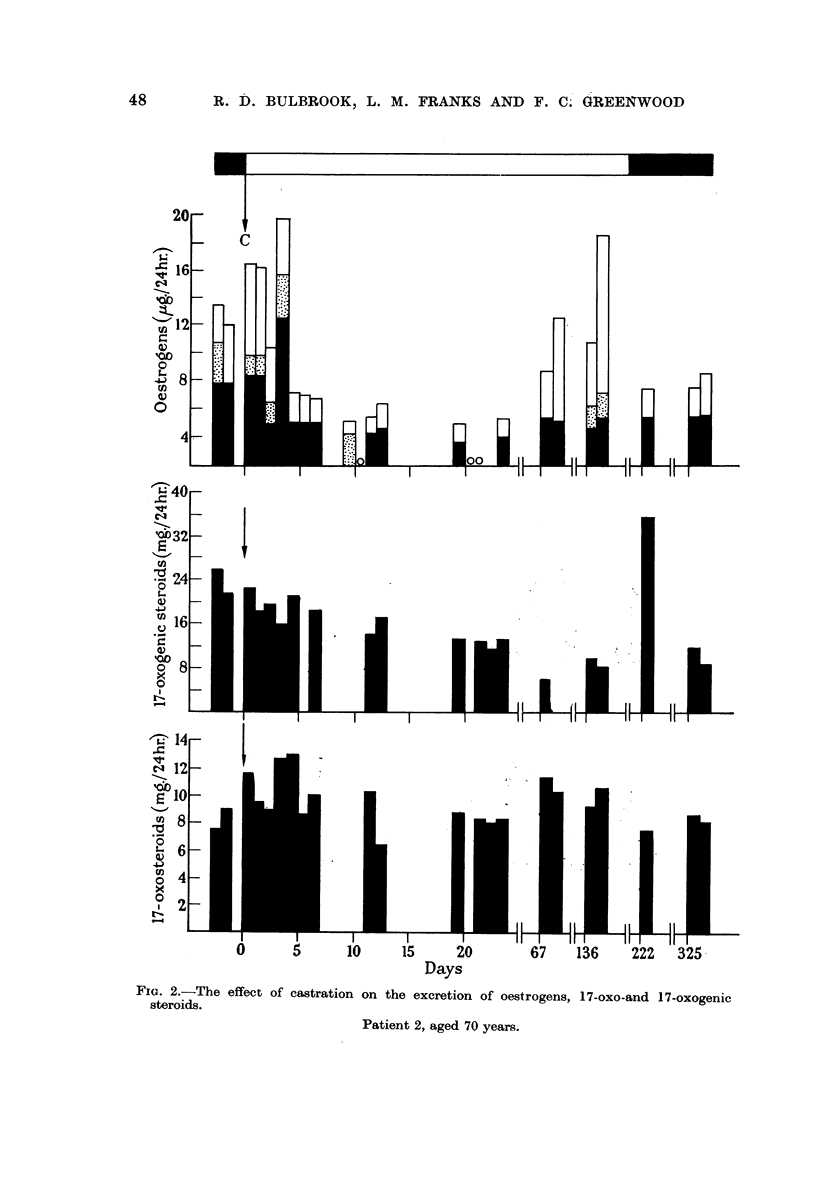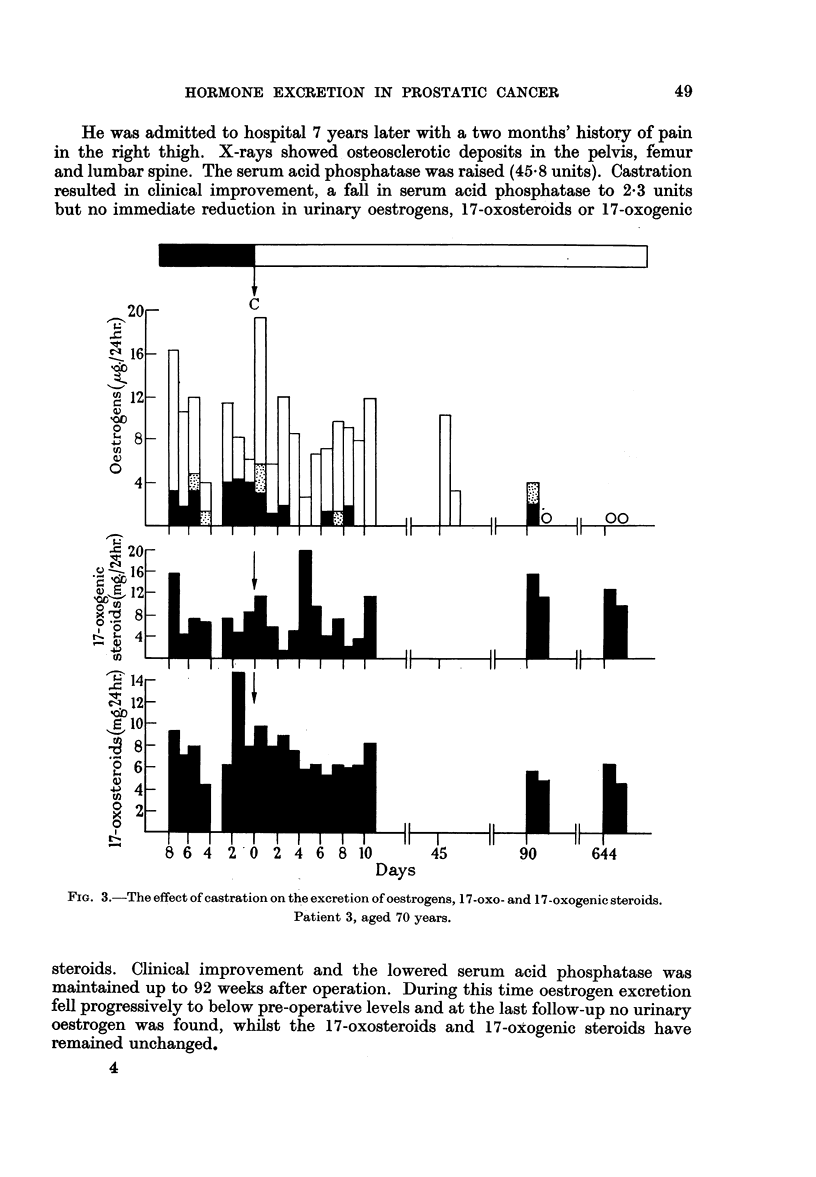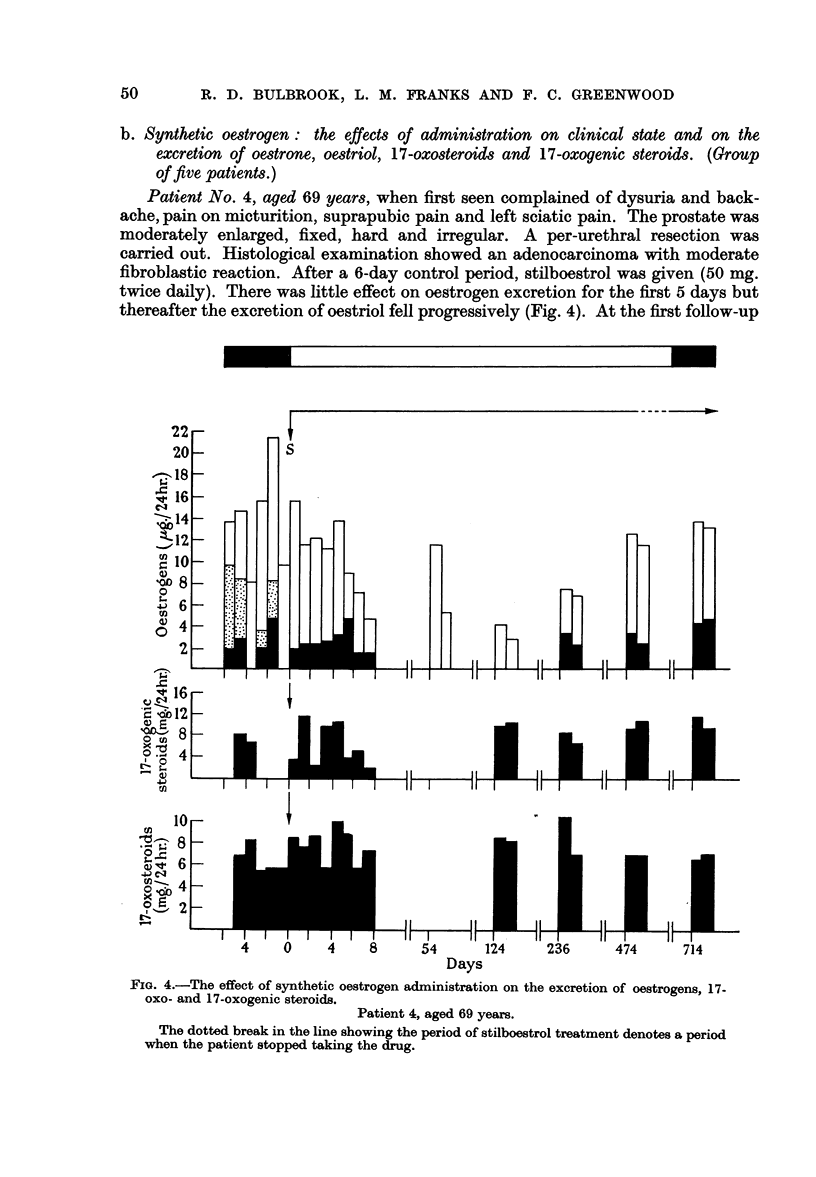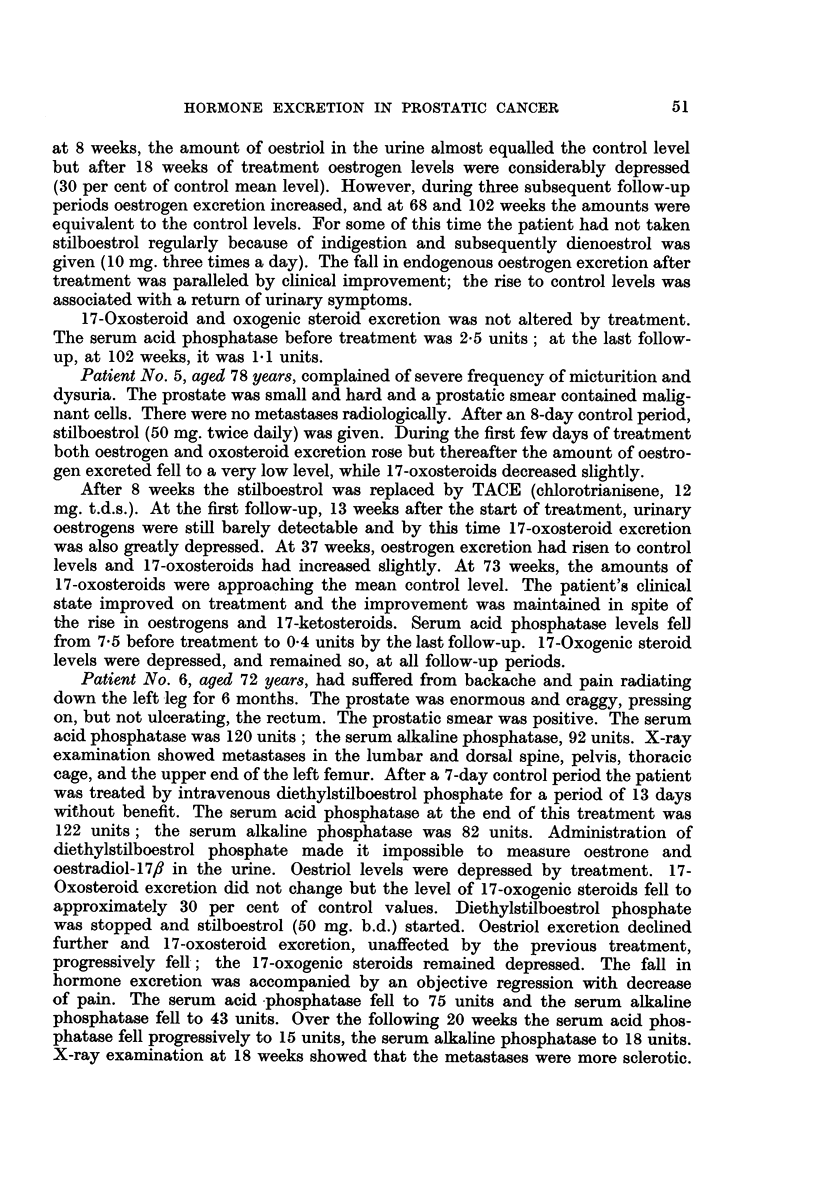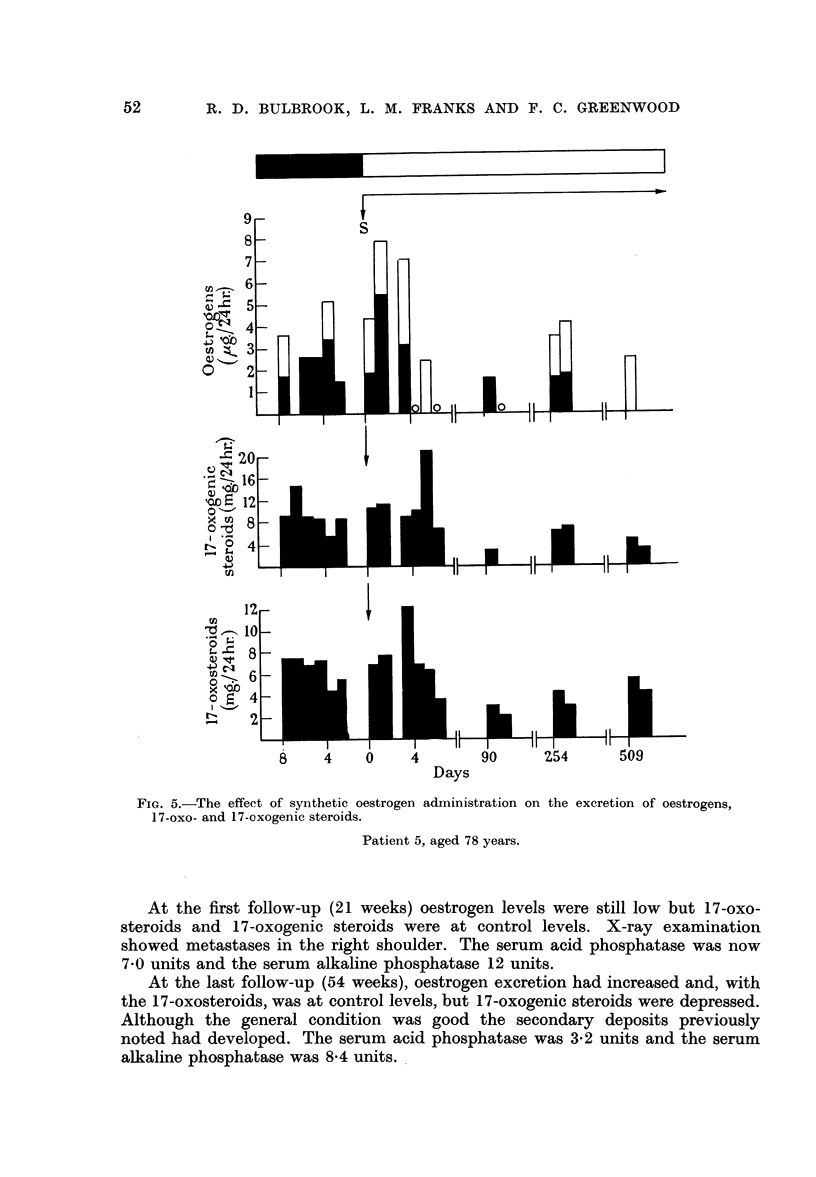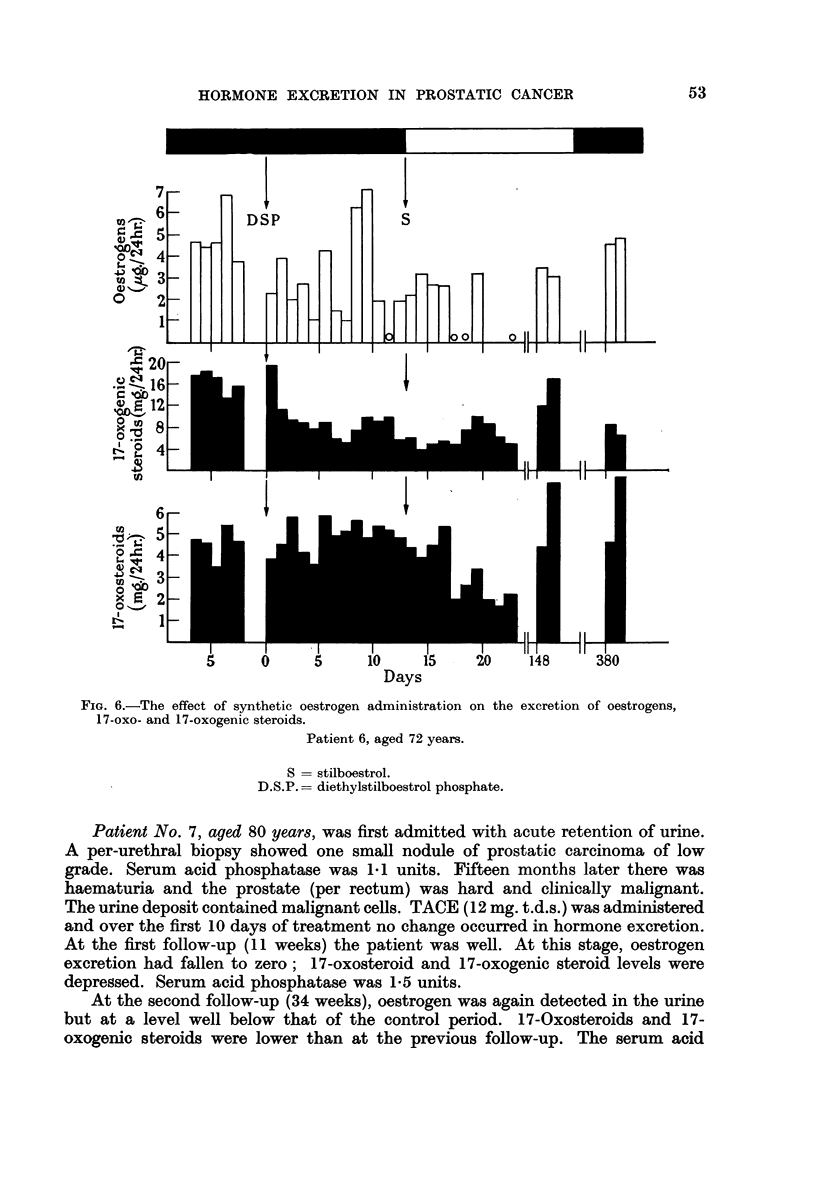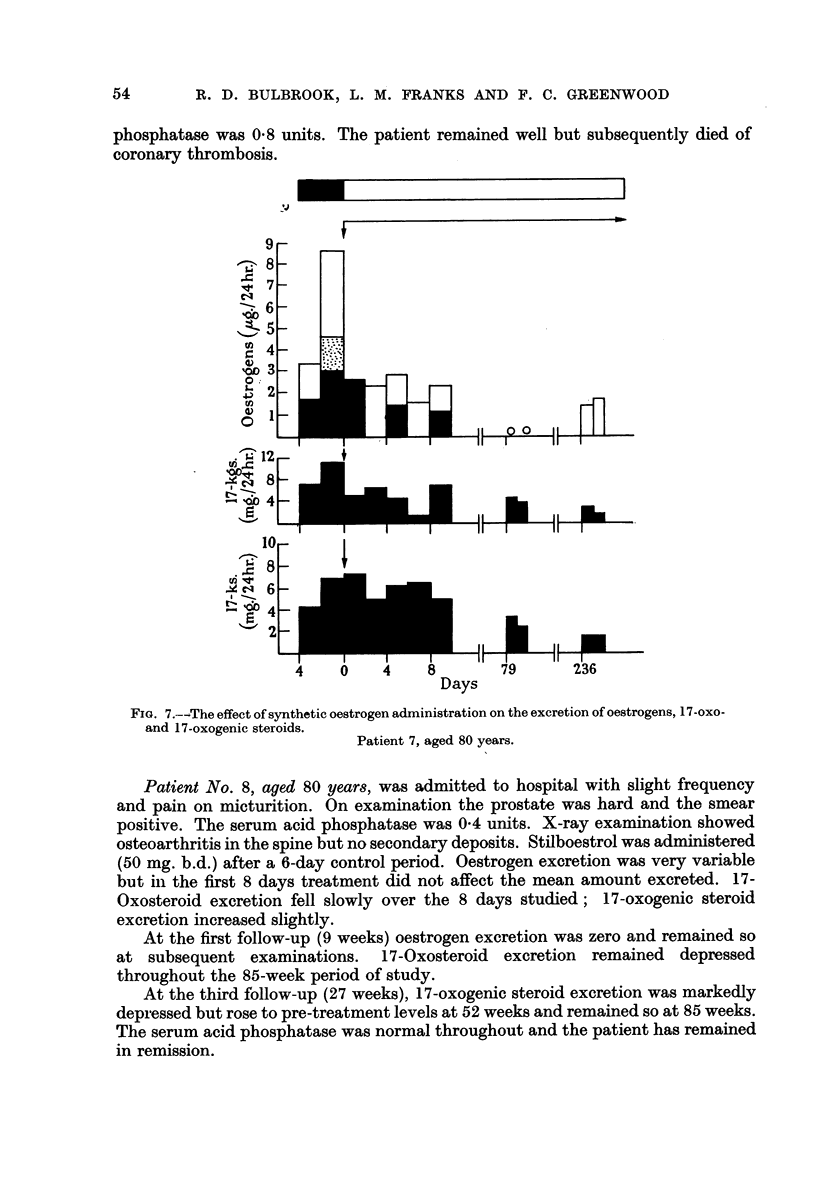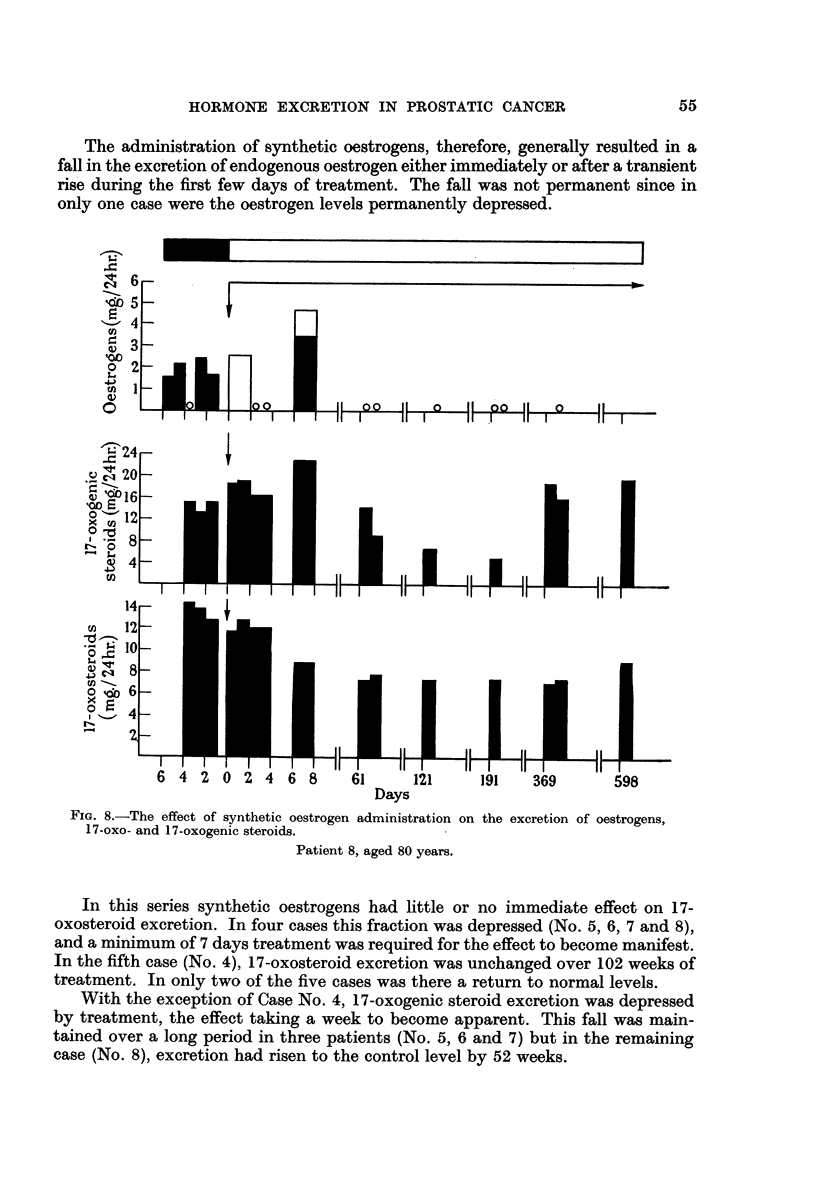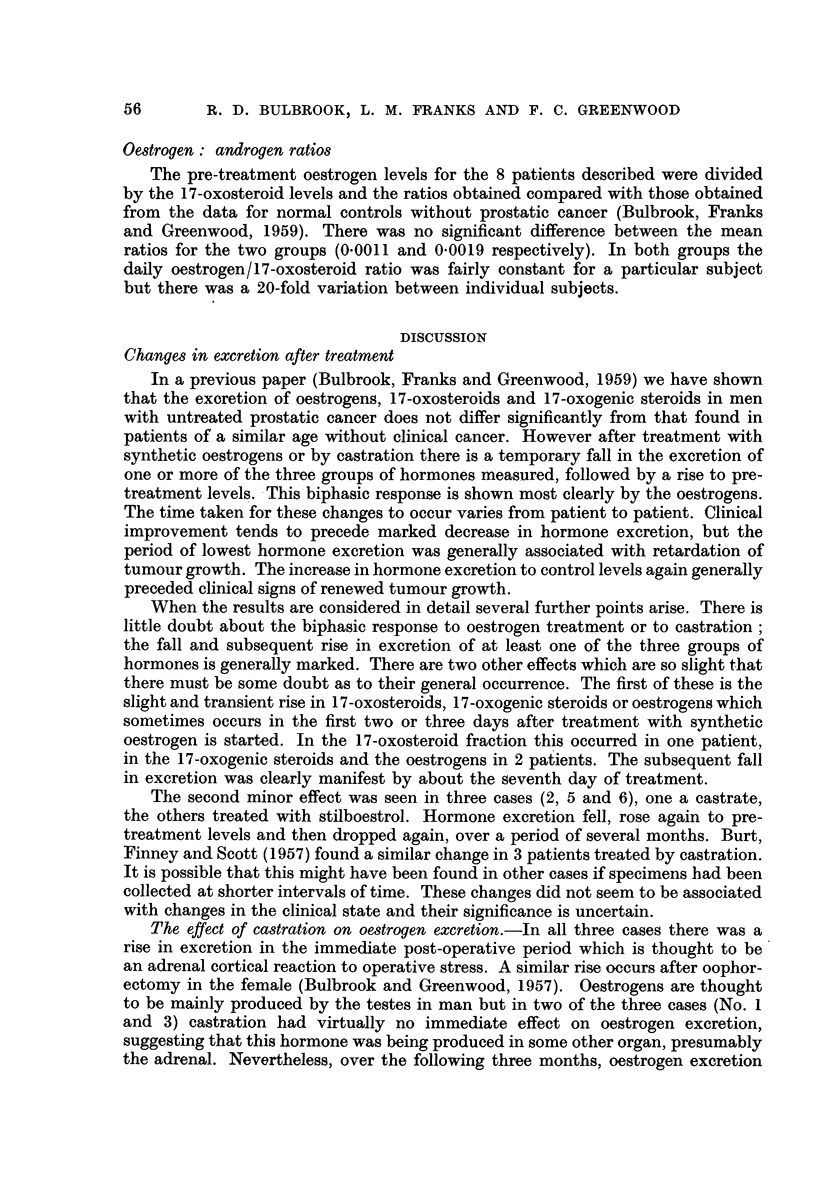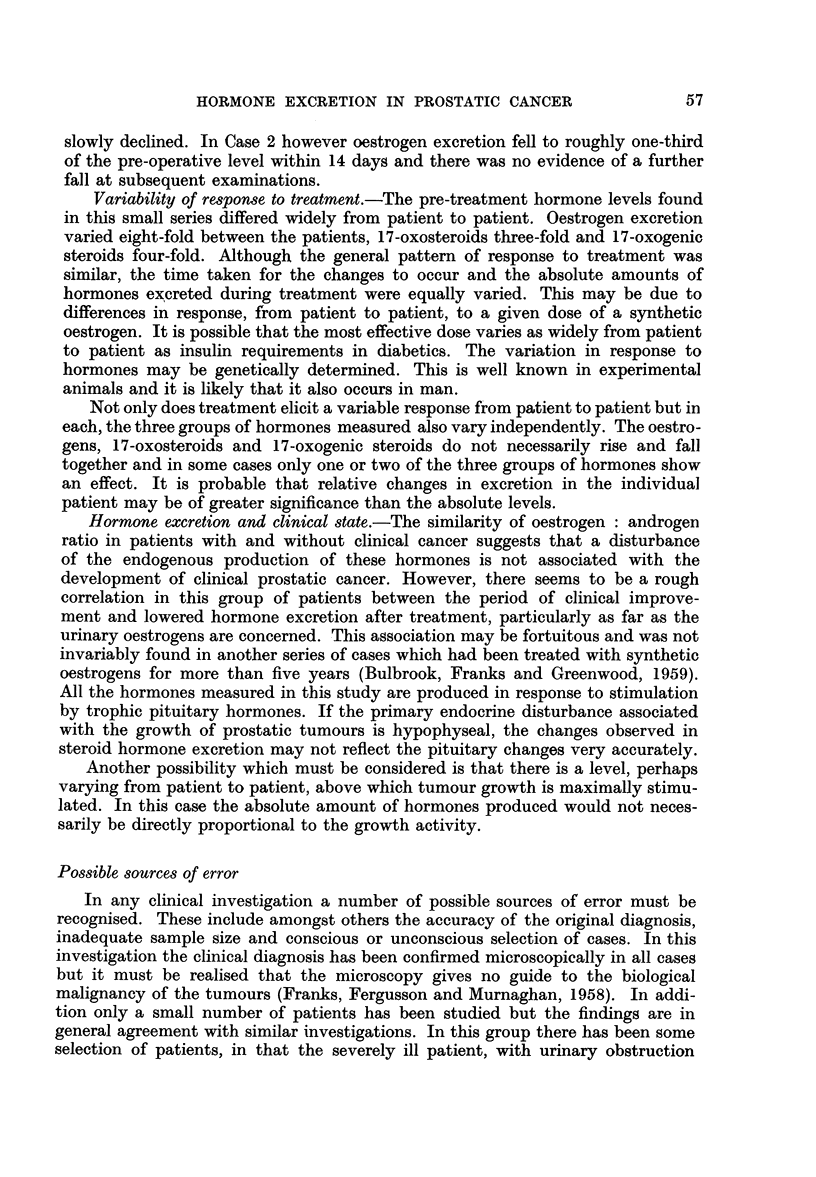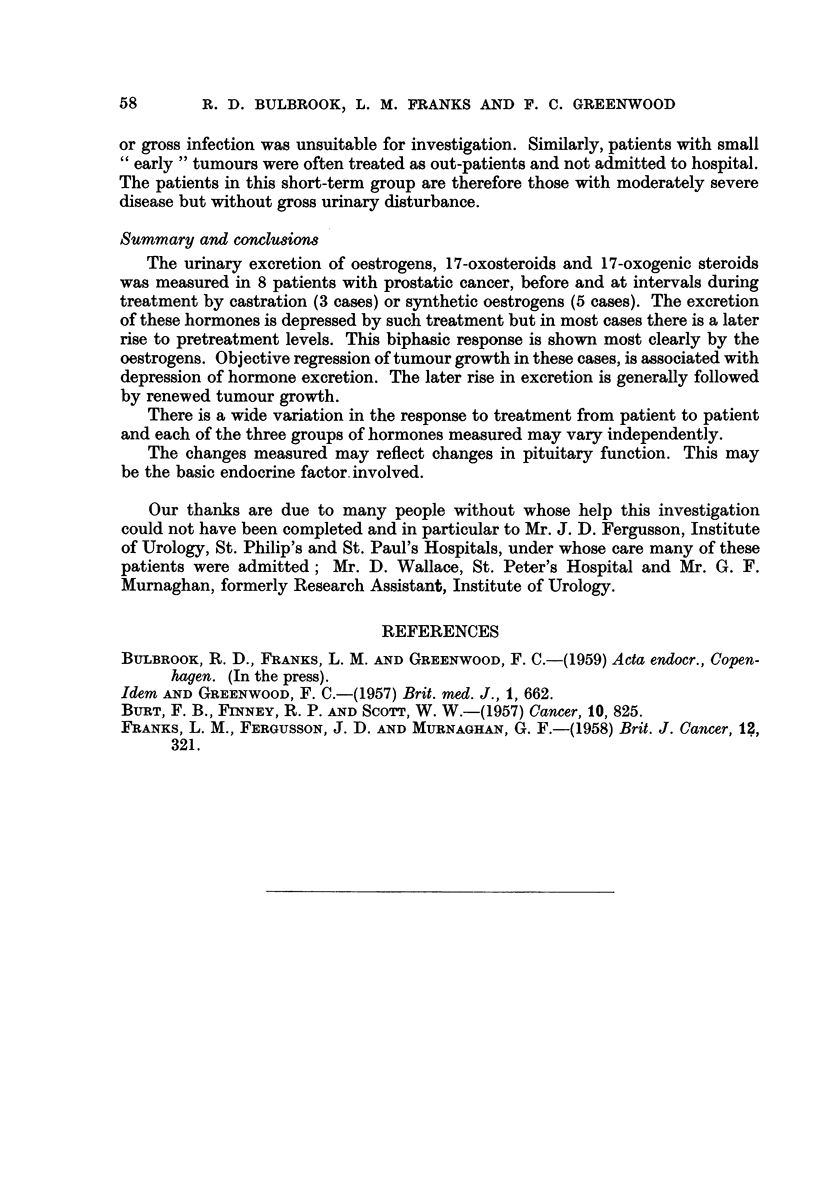# Hormone Excretion in Prostatic Cancer: An Attempt to Correlate Urinary Hormone Excretion and Clinical State

**DOI:** 10.1038/bjc.1959.7

**Published:** 1959-03

**Authors:** R. D. Bulbrook, L. M. Franks, F. C. Greenwood


### HORMONE EXCRETION IN PROSTATIC CANCER: AN ATTEMPT TO CORRELATE URINARY HORMONE EXCRETION AND CLINICAL STATE

R. D. BULBROOK, L. M. FRANKS AND F. C. GREENWOOD

*From the Clinico-pathological Laboratories, Imperial Cancer Research Fund, Lincoln's Inn Fields, London, W.C.2*

Received for publication January 30, 1959

IN a previous paper the excretion of oestrogens, 17-oxosteroids and 17-oxogenic steroids by men with untreated prostatic cancer was compared with that of controls of the same age, and the effects of treatment by synthetic oestrogens or by castration were briefly described (Bulbrook, Franks and Greenwood, 1959). The present paper describes in detail the changes which occur within the first two years of treatment of prostatic cancer by castration or by the administration of synthetic oestrogens and attempts to correlate the clinical effects of such treatment with the changes in hormone excretion in eight patients.

### MATERIAL

Eight patients with prostatic cancer, (aged 66 to 80 years), were admitted to hospital and 24 hour urine specimens collected for three to fifteen days before treatment. The patients were re-admitted to hospital at intervals during treatment for clinical evaluation and urine collections. The clinical diagnosis was confirmed histologically in five cases and cytologically (prostatic smear) in the remaining four. Treatment was by surgical castration (three patients) or by synthetic oestrogens (five patients). The clinical assessment was made without knowledge of the biochemical findings.

### CHEMICAL METHODS

Chemical methods used are as previously described (Bulbrook, Franks and Greenwood, 1959). The effects of synthetic oestrogen treatment on the urinary oestrogen excretion are given for oestrone and oestriol only since the oestradiol-17$\beta$ fraction cannot be measured accurately in urine from these patients. Intravenous diethyl stilboestrol phosphate also interferes with the measurement of oestrone and in one case where this compound was given only oestriol values are reported.

### RESULTS

A brief description of the clinical and biochemical findings is given for each patient. The hormone excretion of the 8 patients is shown graphically in Fig. 1 to 8. Periods of clinical tumour activity are shown by a black bar ; periods of remission by a white bar.

All periods of time have been taken from the day of operation or the start of treatment. The mean hormone excretion in the pre-treatment period—the control level—has been used as the baseline for comparing subsequent hormone determinations during treatment. Age refers to that at operation or at the start of treatment.

a. *Castration : its effects on the clinical state and on the excretion of oestrone, oestradiol-17β, oestriol, 17-oxosteroids and 17-oxogenic steroids.* (*Group of three patients*)

*Patient No.* 1, *aged* 66 *years*, was admitted with incontinence and supra-pubic pain. The prostate was clinically malignant and a prostatic smear contained malignant cells. The serum acid phosphatase (formalin inactivated) was 3·0 units. There were no skeletal metastases on X-ray examination. After a control period of 9 days the patient was treated by castration and per-urethral resection. The resected tissue contained areas of anaplastic carcinoma. The changes in hormone excretion are shown in Fig. 1. In the immediate post-operative period there was a marked rise in excretion of all three groups of steroids measured. This phenomenon has been frequently observed to follow operation and is probably due to an adrenal cortical reaction to stress. After this initial rise the amounts of excreted oestrogens and 17-oxosteroids fell progressively to pre-operative levels. Oestrogens were not detected in the urine 14 weeks after operation, and the 17-oxosteroid excretion was very low. Clinically there was a marked improvement with complete freedom from symptoms. Over the period from 33 to 65 weeks after operation, oestrogen was again detectable in the urine increasing to pre-operative levels and accompanied by an increase in 17-oxosteroid secretion. This period was characterised by the appearance and subsequent development of metastases in the pelvis and spine. When the patient complained of pain, 72 weeks after castration, stilboestrol was given. This was followed by rapid objective and subjective improvement and a second fall in oestrogen and 17-oxosteroid levels. The excretion of 17-oxogenic steroids remained unchanged throughout the period of observation.

*Patient No.* 2, *aged* 70 *years*, was admitted complaining of urinary difficulty for 6 months. Clinically the prostate was malignant and prostatic smears contained malignant cells (confirmed by later biopsy). The serum acid phosphatase was 1·5 units. There were no skeletal metastases on X-ray examination. The patient was castrated and the post-operative hormonal changes are shown in Fig. 2.

A transient rise in oestrogen excretion in the immediate post-operative period was followed by a fall to below the pre-operative levels. Later specimens obtained during follow-up showed a return of excretion to pre-operative values. The 17-oxosteroid levels showed no significant change at operation or in the following 46 weeks, whereas the 17-oxogenic steroids showed a slow fall after operation to low levels but with one exceptionally high value. Clinically the operation resulted in the disappearance of urinary symptoms and an arrest in the tumour growth up to 44 weeks. The re-appearance of mild urinary symptoms was noted some 32 weeks after the return of oestrogen excretion to pre-operative levels, and on examination the prostate was enlarged and hard. This patient died 78 weeks after castration with left ventricular failure. Histologically the prostate removed at autopsy showed many areas of growing tumour.

*Patient No.* 3, *aged* 70 *years*, had a retropubic prostatectomy when 63 years old. Histologically there was an area of prostatic carcinoma in the tissue removed. No treatment was given.

FIG. 1.—The effect of castration on the excretion of oestrogens, 17-oxosteroids and 17-oxogenic steroids.

Patient 1, aged 66 years.

The vertical arrows show the day of treatment.

C = castration.

S = stilboestrol.

Each vertical column gives the result of a duplicate determination on a 24 hour urine specimen. For the oestrogens, the height of the block shows the total amount excreted, subdivided into oestrone (■), oestradiol ([:::]) and oestriol (□).

The horizontal bar at the top of the figure indicates clinical state. The black areas represent tumour growth ; the white areas represent regression. All subsequent figures are plotted in the same way.

FIG. 2.—The effect of castration on the excretion of oestrogens, 17-oxo-and 17-oxogenic steroids.

Patient 2, aged 70 years.

He was admitted to hospital 7 years later with a two months' history of pain in the right thigh. X-rays showed osteosclerotic deposits in the pelvis, femur and lumbar spine. The serum acid phosphatase was raised (45·8 units). Castration resulted in clinical improvement, a fall in serum acid phosphatase to 2·3 units but no immediate reduction in urinary oestrogens, 17-oxosteroids or 17-oxogenic

FIG. 3.—The effect of castration on the excretion of oestrogens, 17-oxo- and 17-oxogenic steroids. Patient 3, aged 70 years.

steroids. Clinical improvement and the lowered serum acid phosphatase was maintained up to 92 weeks after operation. During this time oestrogen excretion fell progressively to below pre-operative levels and at the last follow-up no urinary oestrogen was found, whilst the 17-oxosteroids and 17-oxogenic steroids have remained unchanged.

b. *Synthetic oestrogen : the effects of administration on clinical state and on the excretion of oestrone, oestriol,* 17-*oxosteroids and* 17-*oxogenic steroids.* (*Group of five patients.*)

*Patient No.* 4, *aged* 69 *years,* when first seen complained of dysuria and backache, pain on micturition, suprapubic pain and left sciatic pain. The prostate was moderately enlarged, fixed, hard and irregular. A per-urethral resection was carried out. Histological examination showed an adenocarcinoma with moderate fibroblastic reaction. After a 6-day control period, stilboestrol was given (50 mg. twice daily). There was little effect on oestrogen excretion for the first 5 days but thereafter the excretion of oestriol fell progressively (Fig. 4). At the first follow-up

Fig. 4.—The effect of synthetic oestrogen administration on the excretion of oestrogens, 17-oxo- and 17-oxogenic steroids.

Patient 4, aged 69 years.

The dotted break in the line showing the period of stilboestrol treatment denotes a period when the patient stopped taking the drug.

at 8 weeks, the amount of oestriol in the urine almost equalled the control level but after 18 weeks of treatment oestrogen levels were considerably depressed (30 per cent of control mean level). However, during three subsequent follow-up periods oestrogen excretion increased, and at 68 and 102 weeks the amounts were equivalent to the control levels. For some of this time the patient had not taken stilboestrol regularly because of indigestion and subsequently dienoestrol was given (10 mg. three times a day). The fall in endogenous oestrogen excretion after treatment was paralleled by clinical improvement; the rise to control levels was associated with a return of urinary symptoms.

17-Oxosteroid and oxogenic steroid excretion was not altered by treatment. The serum acid phosphatase before treatment was 2·5 units ; at the last follow-up, at 102 weeks, it was 1·1 units.

*Patient No.* 5, *aged* 78 *years*, complained of severe frequency of micturition and dysuria. The prostate was small and hard and a prostatic smear contained malignant cells. There were no metastases radiologically. After an 8-day control period, stilboestrol (50 mg. twice daily) was given. During the first few days of treatment both oestrogen and oxosteroid excretion rose but thereafter the amount of oestrogen excreted fell to a very low level, while 17-oxosteroids decreased slightly.

After 8 weeks the stilboestrol was replaced by TACE (chlorotrianisene, 12 mg. t.d.s.). At the first follow-up, 13 weeks after the start of treatment, urinary oestrogens were still barely detectable and by this time 17-oxosteroid excretion was also greatly depressed. At 37 weeks, oestrogen excretion had risen to control levels and 17-oxosteroids had increased slightly. At 73 weeks, the amounts of 17-oxosteroids were approaching the mean control level. The patient's clinical state improved on treatment and the improvement was maintained in spite of the rise in oestrogens and 17-ketosteroids. Serum acid phosphatase levels fell from 7·5 before treatment to 0·4 units by the last follow-up. 17-Oxogenic steroid levels were depressed, and remained so, at all follow-up periods.

*Patient No.* 6, *aged* 72 *years*, had suffered from backache and pain radiating down the left leg for 6 months. The prostate was enormous and craggy, pressing on, but not ulcerating, the rectum. The prostatic smear was positive. The serum acid phosphatase was 120 units ; the serum alkaline phosphatase, 92 units. X-ray examination showed metastases in the lumbar and dorsal spine, pelvis, thoracic cage, and the upper end of the left femur. After a 7-day control period the patient was treated by intravenous diethylstilboestrol phosphate for a period of 13 days without benefit. The serum acid phosphatase at the end of this treatment was 122 units ; the serum alkaline phosphatase was 82 units. Administration of diethylstilboestrol phosphate made it impossible to measure oestrone and oestradiol-17$\beta$ in the urine. Oestriol levels were depressed by treatment. 17-Oxosteroid excretion did not change but the level of 17-oxogenic steroids fell to approximately 30 per cent of control values. Diethylstilboestrol phosphate was stopped and stilboestrol (50 mg. b.d.) started. Oestriol excretion declined further and 17-oxosteroid excretion, unaffected by the previous treatment, progressively fell ; the 17-oxogenic steroids remained depressed. The fall in hormone excretion was accompanied by an objective regression with decrease of pain. The serum acid phosphatase fell to 75 units and the serum alkaline phosphatase fell to 43 units. Over the following 20 weeks the serum acid phosphatase fell progressively to 15 units, the serum alkaline phosphatase to 18 units. X-ray examination at 18 weeks showed that the metastases were more sclerotic.

FIG. 5.—The effect of synthetic oestrogen administration on the excretion of oestrogens, 17-oxo- and 17-oxogenic steroids.

Patient 5, aged 78 years.

At the first follow-up (21 weeks) oestrogen levels were still low but 17-oxosteroids and 17-oxogenic steroids were at control levels. X-ray examination showed metastases in the right shoulder. The serum acid phosphatase was now 7·0 units and the serum alkaline phosphatase 12 units.

At the last follow-up (54 weeks), oestrogen excretion had increased and, with the 17-oxosteroids, was at control levels, but 17-oxogenic steroids were depressed. Although the general condition was good the secondary deposits previously noted had developed. The serum acid phosphatase was 3·2 units and the serum alkaline phosphatase was 8·4 units.

Fig. 6.—The effect of synthetic oestrogen administration on the excretion of oestrogens, 17-oxo- and 17-oxogenic steroids.

Patient 6, aged 72 years.

S = stilboestrol.
D.S.P. = diethylstilboestrol phosphate.

*Patient No.* 7, *aged* 80 *years*, was first admitted with acute retention of urine. A per-urethral biopsy showed one small nodule of prostatic carcinoma of low grade. Serum acid phosphatase was 1·1 units. Fifteen months later there was haematuria and the prostate (per rectum) was hard and clinically malignant. The urine deposit contained malignant cells. TACE (12 mg. t.d.s.) was administered and over the first 10 days of treatment no change occurred in hormone excretion. At the first follow-up (11 weeks) the patient was well. At this stage, oestrogen excretion had fallen to zero ; 17-oxosteroid and 17-oxogenic steroid levels were depressed. Serum acid phosphatase was 1·5 units.

At the second follow-up (34 weeks), oestrogen was again detected in the urine but at a level well below that of the control period. 17-Oxosteroids and 17-oxogenic steroids were lower than at the previous follow-up. The serum acid phosphatase was 0·8 units. The patient remained well but subsequently died of coronary thrombosis.

FIG. 7.—The effect of synthetic oestrogen administration on the excretion of oestrogens, 17-oxo- and 17-oxogenic steroids.
Patient 7, aged 80 years.

*Patient No.* 8, *aged* 80 *years*, was admitted to hospital with slight frequency and pain on micturition. On examination the prostate was hard and the smear positive. The serum acid phosphatase was 0·4 units. X-ray examination showed osteoarthritis in the spine but no secondary deposits. Stilboestrol was administered (50 mg. b.d.) after a 6-day control period. Oestrogen excretion was very variable but in the first 8 days treatment did not affect the mean amount excreted. 17-Oxosteroid excretion fell slowly over the 8 days studied ; 17-oxogenic steroid excretion increased slightly.

At the first follow-up (9 weeks) oestrogen excretion was zero and remained so at subsequent examinations. 17-Oxosteroid excretion remained depressed throughout the 85-week period of study.

At the third follow-up (27 weeks), 17-oxogenic steroid excretion was markedly depressed but rose to pre-treatment levels at 52 weeks and remained so at 85 weeks. The serum acid phosphatase was normal throughout and the patient has remained in remission.

The administration of synthetic oestrogens, therefore, generally resulted in a fall in the excretion of endogenous oestrogen either immediately or after a transient rise during the first few days of treatment. The fall was not permanent since in only one case were the oestrogen levels permanently depressed.

FIG. 8.—The effect of synthetic oestrogen administration on the excretion of oestrogens, 17-oxo- and 17-oxogenic steroids.

Patient 8, aged 80 years.

In this series synthetic oestrogens had little or no immediate effect on 17-oxosteroid excretion. In four cases this fraction was depressed (No. 5, 6, 7 and 8), and a minimum of 7 days treatment was required for the effect to become manifest. In the fifth case (No. 4), 17-oxosteroid excretion was unchanged over 102 weeks of treatment. In only two of the five cases was there a return to normal levels.

With the exception of Case No. 4, 17-oxogenic steroid excretion was depressed by treatment, the effect taking a week to become apparent. This fall was maintained over a long period in three patients (No. 5, 6 and 7) but in the remaining case (No. 8), excretion had risen to the control level by 52 weeks.

*Oestrogen : androgen ratios*

The pre-treatment oestrogen levels for the 8 patients described were divided by the 17-oxosteroid levels and the ratios obtained compared with those obtained from the data for normal controls without prostatic cancer (Bulbrook, Franks and Greenwood, 1959). There was no significant difference between the mean ratios for the two groups (0·0011 and 0·0019 respectively). In both groups the daily oestrogen/17-oxosteroid ratio was fairly constant for a particular subject but there was a 20-fold variation between individual subjects.

### DISCUSSION

*Changes in excretion after treatment*

In a previous paper (Bulbrook, Franks and Greenwood, 1959) we have shown that the excretion of oestrogens, 17-oxosteroids and 17-oxogenic steroids in men with untreated prostatic cancer does not differ significantly from that found in patients of a similar age without clinical cancer. However after treatment with synthetic oestrogens or by castration there is a temporary fall in the excretion of one or more of the three groups of hormones measured, followed by a rise to pre-treatment levels. This biphasic response is shown most clearly by the oestrogens. The time taken for these changes to occur varies from patient to patient. Clinical improvement tends to precede marked decrease in hormone excretion, but the period of lowest hormone excretion was generally associated with retardation of tumour growth. The increase in hormone excretion to control levels again generally preceded clinical signs of renewed tumour growth.

When the results are considered in detail several further points arise. There is little doubt about the biphasic response to oestrogen treatment or to castration ; the fall and subsequent rise in excretion of at least one of the three groups of hormones is generally marked. There are two other effects which are so slight that there must be some doubt as to their general occurrence. The first of these is the slight and transient rise in 17-oxosteroids, 17-oxogenic steroids or oestrogens which sometimes occurs in the first two or three days after treatment with synthetic oestrogen is started. In the 17-oxosteroid fraction this occurred in one patient, in the 17-oxogenic steroids and the oestrogens in 2 patients. The subsequent fall in excretion was clearly manifest by about the seventh day of treatment.

The second minor effect was seen in three cases (2, 5 and 6), one a castrate, the others treated with stilboestrol. Hormone excretion fell, rose again to pre-treatment levels and then dropped again, over a period of several months. Burt, Finney and Scott (1957) found a similar change in 3 patients treated by castration. It is possible that this might have been found in other cases if specimens had been collected at shorter intervals of time. These changes did not seem to be associated with changes in the clinical state and their significance is uncertain.

*The effect of castration on oestrogen excretion.*—In all three cases there was a rise in excretion in the immediate post-operative period which is thought to be an adrenal cortical reaction to operative stress. A similar rise occurs after oophorectomy in the female (Bulbrook and Greenwood, 1957). Oestrogens are thought to be mainly produced by the testes in man but in two of the three cases (No. 1 and 3) castration had virtually no immediate effect on oestrogen excretion, suggesting that this hormone was being produced in some other organ, presumably the adrenal. Nevertheless, over the following three months, oestrogen excretion slowly declined. In Case 2 however oestrogen excretion fell to roughly one-third of the pre-operative level within 14 days and there was no evidence of a further fall at subsequent examinations.

*Variability of response to treatment.*—The pre-treatment hormone levels found in this small series differed widely from patient to patient. Oestrogen excretion varied eight-fold between the patients, 17-oxosteroids three-fold and 17-oxogenic steroids four-fold. Although the general pattern of response to treatment was similar, the time taken for the changes to occur and the absolute amounts of hormones excreted during treatment were equally varied. This may be due to differences in response, from patient to patient, to a given dose of a synthetic oestrogen. It is possible that the most effective dose varies as widely from patient to patient as insulin requirements in diabetics. The variation in response to hormones may be genetically determined. This is well known in experimental animals and it is likely that it also occurs in man.

Not only does treatment elicit a variable response from patient to patient but in each, the three groups of hormones measured also vary independently. The oestrogens, 17-oxosteroids and 17-oxogenic steroids do not necessarily rise and fall together and in some cases only one or two of the three groups of hormones show an effect. It is probable that relative changes in excretion in the individual patient may be of greater significance than the absolute levels.

*Hormone excretion and clinical state.*—The similarity of oestrogen : androgen ratio in patients with and without clinical cancer suggests that a disturbance of the endogenous production of these hormones is not associated with the development of clinical prostatic cancer. However, there seems to be a rough correlation in this group of patients between the period of clinical improvement and lowered hormone excretion after treatment, particularly as far as the urinary oestrogens are concerned. This association may be fortuitous and was not invariably found in another series of cases which had been treated with synthetic oestrogens for more than five years (Bulbrook, Franks and Greenwood, 1959). All the hormones measured in this study are produced in response to stimulation by trophic pituitary hormones. If the primary endocrine disturbance associated with the growth of prostatic tumours is hypophyseal, the changes observed in steroid hormone excretion may not reflect the pituitary changes very accurately.

Another possibility which must be considered is that there is a level, perhaps varying from patient to patient, above which tumour growth is maximally stimulated. In this case the absolute amount of hormones produced would not necessarily be directly proportional to the growth activity.

### *Possible sources of error*

In any clinical investigation a number of possible sources of error must be recognised. These include amongst others the accuracy of the original diagnosis, inadequate sample size and conscious or unconscious selection of cases. In this investigation the clinical diagnosis has been confirmed microscopically in all cases but it must be realised that the microscopy gives no guide to the biological malignancy of the tumours (Franks, Fergusson and Murnaghan, 1958). In addition only a small number of patients has been studied but the findings are in general agreement with similar investigations. In this group there has been some selection of patients, in that the severely ill patient, with urinary obstruction or gross infection was unsuitable for investigation. Similarly, patients with small "early" tumours were often treated as out-patients and not admitted to hospital. The patients in this short-term group are therefore those with moderately severe disease but without gross urinary disturbance.

*Summary and conclusions*

The urinary excretion of oestrogens, 17-oxosteroids and 17-oxogenic steroids was measured in 8 patients with prostatic cancer, before and at intervals during treatment by castration (3 cases) or synthetic oestrogens (5 cases). The excretion of these hormones is depressed by such treatment but in most cases there is a later rise to pretreatment levels. This biphasic response is shown most clearly by the oestrogens. Objective regression of tumour growth in these cases, is associated with depression of hormone excretion. The later rise in excretion is generally followed by renewed tumour growth.

There is a wide variation in the response to treatment from patient to patient and each of the three groups of hormones measured may vary independently.

The changes measured may reflect changes in pituitary function. This may be the basic endocrine factor involved.

Our thanks are due to many people without whose help this investigation could not have been completed and in particular to Mr. J. D. Fergusson, Institute of Urology, St. Philip's and St. Paul's Hospitals, under whose care many of these patients were admitted ; Mr. D. Wallace, St. Peter's Hospital and Mr. G. F. Murnaghan, formerly Research Assistant, Institute of Urology.

### REFERENCES

BULBROOK, R. D., FRANKS, L. M. AND GREENWOOD, F. C.—(1959) *Acta endocr., Copenhagen.* (In the press).

*Idem* AND GREENWOOD, F. C.—(1957) *Brit. med. J.*, **1**, 662.

BURT, F. B., FINNEY, R. P. AND SCOTT, W. W.—(1957) *Cancer*, **10**, 825.

FRANKS, L. M., FERGUSSON, J. D. AND MURNAGHAN, G. F.—(1958) *Brit. J. Cancer*, **12**, 321.